# Transforming Porous Membranes into Dual‐Gradient Janus Structures by Directional Asymmetric Modification

**DOI:** 10.1002/smtd.202500839

**Published:** 2025-08-07

**Authors:** Jaehyung Jeon, Heeseon Choi, Jinseung Bae, Gwang Myeong Seo, Jeonghun Han, Hogyun Park, Sungsu Park

**Affiliations:** ^1^ School of Mechanical Engineering Sungkyunkwan University (SKKU) Seoburo 2066, Jangan‐gu Suwon 16419 South Korea

**Keywords:** Janus membranes, plasma separation, porosity gradient, self‐pumping, wettability gradient

## Abstract

Janus membranes are essential in applications such as water purification, biomedical diagnostics, and energy systems owing to their tunable pore structures and surface properties, which enable efficient fluid and ion transport. Here, a scalable strategy is presented for transforming conventional porous membranes into dual‐gradient Janus structures through a parylene–plasma–porous (PPP) treatment that enables simultaneous and aligned control of porosity and wettability within a single porous substrate. This process integrates asymmetric parylene C deposition and O_2_ plasma treatment to create vertically asymmetric structures and surface wettability, resulting in gravity‐independent, self‐pumping fluid transport driven by capillary forces. The dual gradients are validated through morphological and chemical characterization, and the versatility of the method is further demonstrated by its successful application to both glass fiber (GF) and cellulose membranes. Functionally, the resulting Janus membrane achieves efficient plasma separation from microliter‐scale whole blood, exhibiting low hemolysis and high protein recovery without external power. This approach provides a broadly applicable platform for fabricating functional gradient membranes tailored for advanced microfluidic and diagnostic systems.

## Graphical abstract

This study reports a parylene–plasma–porous (PPP) treatment that directionally transforms conventional porous membranes into dual‐gradient Janus structures. By aligning porosity and wettability gradients through asymmetric chemical‐vapor‐deposition (CVD) and localized O_2_ plasma treatment, the resulting membrane enables gravity‐independent self‐pumping and blood plasma separation. This scalable, post‐treatment method serves as a versatile platform for next‐generation membrane design in point‐of‐care diagnostics.

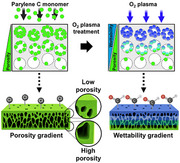

## 1 Introduction

Conventional porous membranes often suffer from structural limitations, such as uniform wettability and porosity, which restrict their functionality in applications requiring directional fluid transport, selective separation, or gradient‐driven flow. These shortcomings hinder their performance in complex environments such as microfluidic systems, biomedical diagnostics, and environmental filtration. Recent research has attempted to overcome the limitations of conventional porous membranes by developing Janus membranes, where different layers of the membrane exhibit distinct wettability or porosity characteristics.^[^ ^1^, ^2^, ^3^, ^4^, ^5^ ^]^ These membranes are often fabricated by physical layering different materials or using blow spinning techniques to join separate porous structures.^[^ ^3^, ^6^, ^7^ ^]^ However, these approaches still rely on discrete layering, which can result in inconsistent interfacial adhesion, delamination, and increased fabrication complexity. A truly effective 3D gradient‐controlled porous membrane could overcome these limitations by providing a single, seamlessly integrated structure with continuous functionality.

Parylene C, a versatile polymer composed of repeated units of poly(p‐chloro‐xylylene), has been extensively used to coat porous membranes due to its unique properties, including chemical inertness, biocompatibility, and its ability to conformally coat complex structures.^[^ ^8^, ^9^, ^10^ ^]^ Its chemical structure features benzene rings and chlorine substituents, which contribute to its excellent barrier properties and inherent hydrophobicity.^[^ ^11^, ^12^ ^]^ The hydrophobic nature of parylene C arises from its stable, non‐polar carbon backbone and the presence of the chlorine group, which reduces the surface energy and limits water affinity. This polymer has enabled advancements across various fields, particularly by enhancing membrane durability, tuning surface wettability, and protecting against chemical or biological degradation.^[^ ^12^, ^13^, ^14^, ^15^, ^16^ ^]^ However, conventional parylene C treatments typically result in uniform coatings that lack the spatial control necessary for creating functional gradients, such as porosity or wettability patterns, in three dimensions (3D).

In this study, we present a strategy to convert conventional porous membranes into dual‐gradient Janus structures via a parylene–plasma–porous (PPP) treatment. By combining asymmetric chemical vapor deposition (CVD) of parylene C with selective O_2_ plasma treatment, we generate aligned porosity and wettability gradients in a single membrane to drive self‐pumping capillary flow independent of gravity. First, parylene C deposits predominantly on the top face—thickening fibers and reducing porosity—while the bottom retains high porosity. Next, selective O_2_ plasma converts that same face to hydrophilic (–COOH/–CHO), yielding a matched wettability gradient. Together, these dual gradients power gravity‐independent fluid transport in a robust, solvent‐free, room‐temperature (RT) process. We confirmed the dual‐gradient architecture by using field‐emission scanning electron microscopy (FE‐SEM), energy‐dispersive X‐ray spectroscopy (EDS), and X‐ray photoelectron spectroscopy (XPS), and demonstrated broad applicability by extending the method to cellulose membranes. Functionally, our Janus membranes achieved efficient, self‐driven plasma separation from microliter‐scale blood samples, underscoring their structural tunability and practical relevance.

## 2 Results and Discussion

### 2.1 Fabrication of Dual‐Gradient Janus Membrane

To transform a conventional porous membrane into a dual‐gradient Janus membrane, we employed a PPP treatment based on a directional modification strategy that combines asymmetric CVD of parylene C with selective O_2_ plasma treatment. Here, “directional modification” refers to applying treatments in a directionally confined manner so that they modify only one face of the membrane, creating a gradient across its depth.

**Figure 1:**
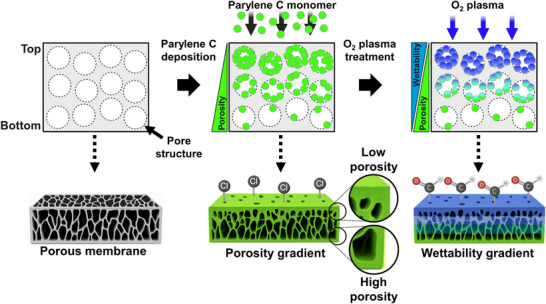
Schematic of the PPP treatment process. Asymmetric CVD of parylene C creates a porosity gradient by depositing more material on the top surface. Subsequent O_2_ plasma treatment selectively modifies the exposed top surface, forming a wettability gradient that aligns with the porosity gradient. The top‐row cross‐sectional schematics use stylized circles to represent disordered pores, while the 3D cutaway below illustrates the actual membrane microstructure.

In brief, asymmetric CVD of parylene C establishes a porosity gradient by accumulating to a greater extent on the top surface, resulting in lower porosity and thicker fibers, whereas the bottom retains higher porosity and thinner fibers due to limited diffusion of parylene C monomers (**Figure** **1**). This occurs through a vapor‐phase polymerization mechanism, in which parylene C dimers are thermally cracked into reactive monomers that deposit conformally onto the exposed membrane surface and penetrate through interconnected pores. Because the membrane's bottom side is sealed during deposition, monomer diffusion occurs preferentially from the top, leading to a gradient in polymer accumulation throughout the membrane depth. This increases fiber thickness progressively from bottom to top, yielding a structural porosity gradient without collapsing the original fiber network. Subsequent directional O_2_ plasma treatment selectively introduces hydrophilic functional groups (e.g., aldehydes) to the top surface, forming a wettability gradient aligned with the porosity gradient. O_2_ plasma modifies the parylene C surface by cleaving the C–Cl bonds and introducing oxygen‐containing functional groups (─CHO and ─COO^−^), thereby enhancing the surface hydrophilicity.^[^ ^17^, ^18^ ^]^

Our directional modification approach converts existing porous membranes into Janus structures through spatially controlled post‐treatment, without the need for multilayer bonding or spinning‐based fabrication steps. This method is material agnostic, scalable, and compatible with room temperature (RT) processing, making it well‐suited for large‐area manufacturing and integration into various membrane‐based platforms.

### 2.2 Characterization of Dual‐Gradient Janus Membrane

**Figure 2:**
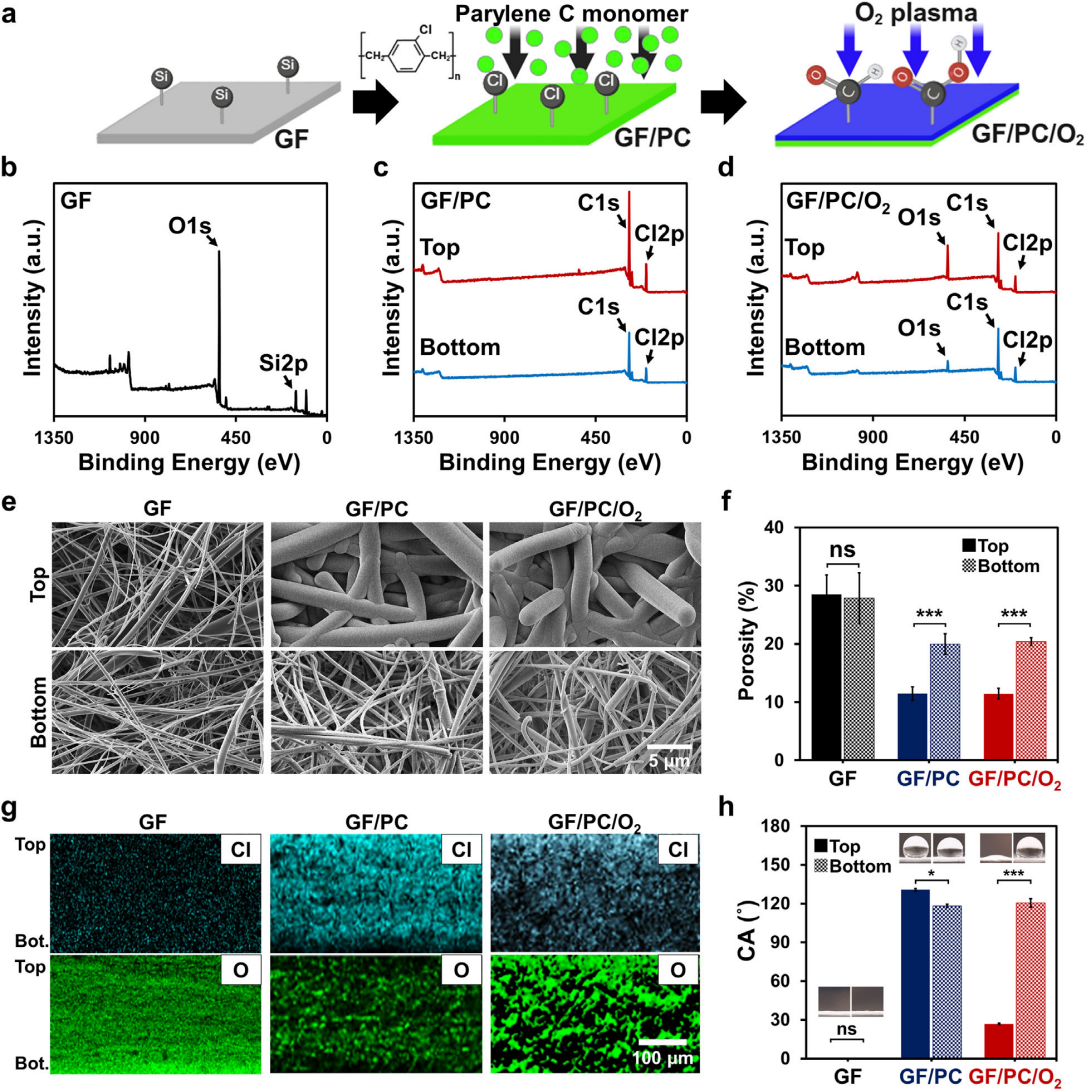
Characterization of a dual‐gradient Janus membrane. a) Schematic of the PPP treatment process illustrating stepwise surface modification of the GF. Asymmetric CVD of parylene C introduces hydrophobic Cl groups, forming a porosity gradient. Subsequent O_2_ plasma treatment oxidizes the surface of parylene C, generating hydrophilic functionalities (e.g., –COOH, –CHO) and establishing a wettability gradient aligned with the porosity profile. b–d) XPS survey spectra of untreated GF, parylene C‐coated GF (GF/PC), and GF/PC after O_2_ plasma treatment (GF/PC/O_2_), showing elemental transitions at each fabrication stage. e) FE‐SEM (JSM‐7500F, JEOL Ltd.; ×5000) images of the top and bottom surfaces of GF, GF/PC, and GF/PC/O_2_, revealing changes in the fiber morphology and thickness. f) Porosity analysis of the top and bottom surfaces of the GF, GF/PC, and GF/PC/O_2_ (*n* = 3, Student's *t*‐test: ns *p* >0.05, ^***^ *p* <0.001). g) EDS elemental mapping of Cl and O across the membrane cross‐section for GF, GF/PC, and GF/PC/O_2_, depicting asymmetric parylene C deposition and selective surface oxidation. h) Comparison of water contact angle (CA) between the top and bottom surfaces of each membrane (*n* = 3, Student's *t*‐test: ns *p* >0.05, ^*^ *p* <0.05, ^***^ *p* <0.001).

Asymmetric CVD of parylene C established a porosity gradient by accumulating more extensively on the top surface of the GF, introducing Cl‐containing hydrophobic groups, and resulting in reduced porosity and thicker fibers. In contrast, the bottom surface retained higher porosity and thinner fibers due to limited diffusion of parylene C monomers through the membrane network (**Figure** **2**a). Subsequent O_2_ plasma treatment selectively oxidized the parylene coated top surface, replacing Cl groups with oxygen‐containing hydrophilic functionalities (e.g., –COOH, –CHO), thereby forming a wettability gradient aligned with the porosity gradient.

XPS analysis was performed to monitor elemental changes on the top and bottom surfaces, probing a depth of ≈10 nm at each modification step. Chlorine (Cl), an intrinsic element of parylene C, indicates coating deposition, whereas oxygen (O) reflects the hydrophilic surface modification from the O_2_ plasma treatment. The XPS survey spectra of the untreated GF showed a silicon (Si 2p) peak at 103 eV, characteristic of the SiO_2_‐based surface (Figure 2b). After the parylene C deposition (GF/PC), a Cl 2p peak appeared at 200 eV on both the top and bottom surfaces, whereas the Si peak disappeared (Figure 2c). The Cl 2p intensity was ≈2.8 times higher at the top surface, confirming asymmetric parylene C deposition. Following the O_2_ plasma treatment (GF/PC/O_2_), a strong O 1s peak emerged at 532 eV, with the top surface having approximately two‐fold higher intensity than the bottom—indicating successful and asymmetric surface functionalization (Figure 2d). The observed decrease in Cl 2p intensity after O_2_ plasma treatment was attributed to the conversion of C–Cl groups of parylene C into oxygen‐containing hydrophilic functionalities (e.g., –CHO or –COOH), consistent with the proposed surface modification mechanism.

FE‐SEM imaging (Figure 2e) revealed the evolution of fiber structure across the modification steps, confirming the formation of a porosity gradient. Untreated GF exhibited uniform, thin fibers and an open porous structure. After parylene C deposition (GF/PC), the top surface showed thicker and denser fibers due to directional accumulation, whereas the bottom surface retained thinner fibers, indicating successful gradient formation. Following the O_2_ plasma treatment (GF/PC/O_2_), the overall fiber morphology remained unchanged, confirming the structural stability of the gradient. These observations were supported by porosity measurements (Figure 2f), where the untreated GF showed uniformly high porosity (≈28%), while GF/PC exhibited reduced porosity at the top surface (11%) and moderately high porosity at the bottom (20%). The porosity distribution remained consistent after the O_2_ plasma treatment, validating that the PPP treatment effectively creates and maintains a stable porosity gradient.

Cross‐sectional EDS mapping was conducted to confirm the formation of porosity and wettability gradients across the membrane (Figure 2g), examining elemental distributions throughout the full membrane depth of ≈260 µm. The Cl distribution was analyzed to assess the parylene C deposition, whereas the O distribution indicated the introduction of hydrophilic surface groups. In untreated GF, no Cl signal was detected, and O was uniformly distributed across the membrane, reflecting the inherent composition of the GF. In GF/PC, the Cl intensity was the highest at the top surface and gradually decreased toward the bottom, confirming asymmetric parylene C deposition. Following the treatment (GF/PC/O_2_), the Cl profile, in which chlorine was more concentrated on the top side than the bottom, was still observed. However, the overall Cl intensity decreased, suggesting chemical modification of the parylene C layer. In contrast, the O intensity increased significantly at the top surface with only a minor increase at the bottom, verifying that oxygen‐containing functional groups were introduced selectively, forming a wettability gradient aligned with the pre‐existing porosity gradient.

These elemental differences influence surface wettability by promoting hydrophobicity in Cl rich regions and enhancing hydrophilicity through oxygen‐containing polar groups such as hydroxyl, carbonyl, and carboxyl.^[^ ^14^, ^18^ ^]^ The contact angle (CA) measurements support these trends (Figure 2h). The GF exhibited complete hydrophilicity (CA: ≈0°) on both surfaces, whereas GF/PC became hydrophobic (top: 130°, bottom: 118°). After O_2_ plasma treatment, the top surface of GF/PC/O_2_ became hydrophilic (CA: 26°), whereas the bottom remained hydrophobic (CA: 120°). Together with the XPS and EDS data, these results confirm that the PPP treatment successfully induces dual gradients in porosity and wettability.

### 2.3 Effect of Level of Parylene C Deposition on Porosity Gradient Formation

**Figure 3:**
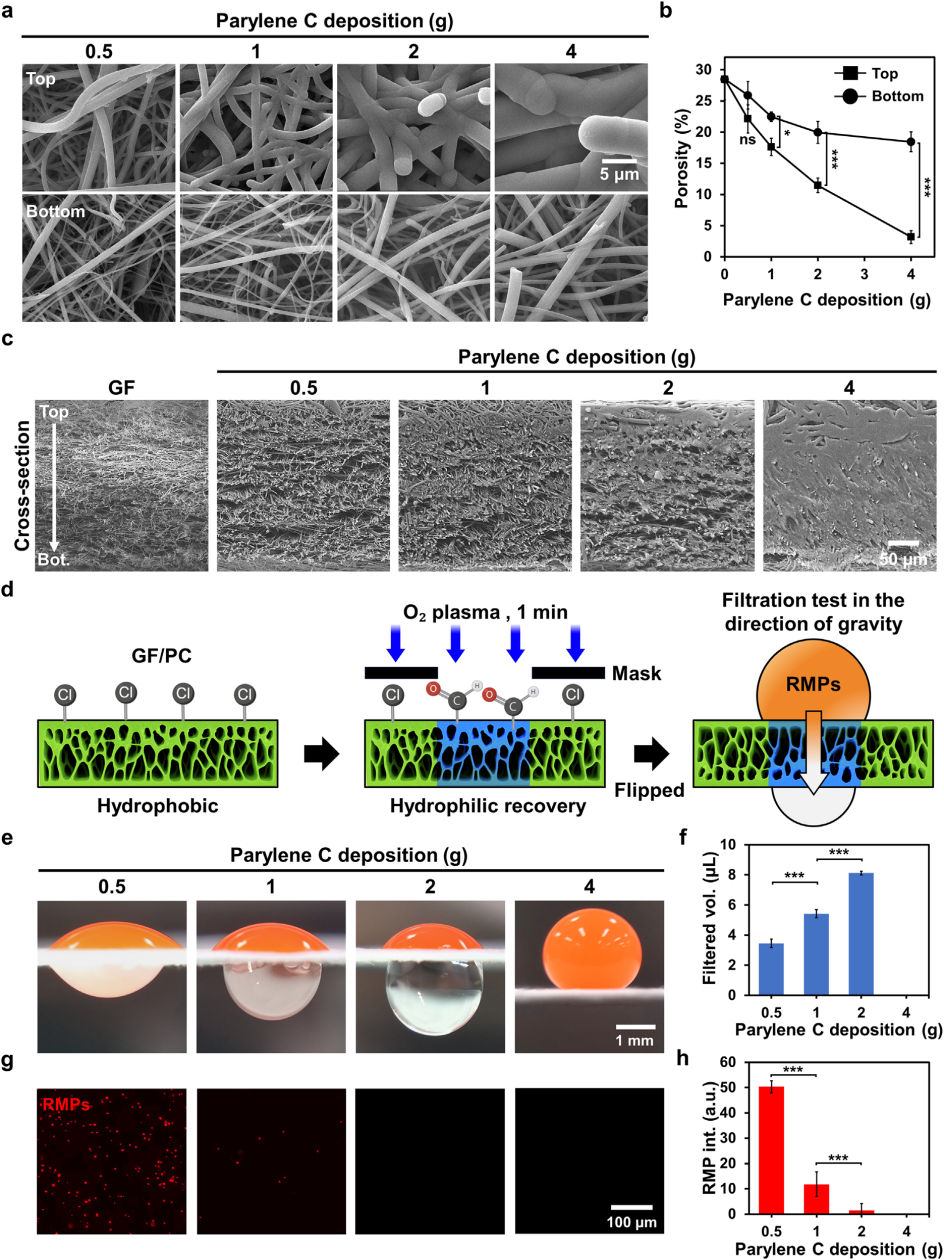
Characterization of porosity gradient formed by varying the parylene C deposition. a) SEM images (×3000) of the top and bottom surfaces of GF/PC membranes with increasing parylene C deposition conditions (0.5–4 g). b) Quantitative porosity analysis of the of top and bottom surfaces of GF/PC membranes as a function of parylene C deposition (*n* = 3, Student's *t*‐test: ns *p* >0.05, ^*^ *p* <0.05, ^***^ *p* <0.001). c) Cross‐sectional SEM images (×700) of original GF and GF/PC membranes with increasing parylene C deposition conditions (0.5–4 g). d) Schematic of the filtration test using GF/PC membranes with red fluorescent microparticles (RMPs, 1–5 µm in diameter). Hydrophilic paths (2.5 mm in diameter) were generated by O_2_ plasma treatment for 1 min through a circular mask. The membranes were then flipped, and a 10 µL droplet of RMP‐containing PBS was applied to the surface (larger porosity side) for gravity‐driven filtration. e) Optical images of droplets on GF/PC membranes 8 min after loading, with increasing parylene C deposition (0.5–4 g). f) Quantitative analysis of the filtered volume after 8 min for membranes with varying levels of parylene C deposition (*n* = 3, Student's *t*‐test: ^***^ *p* <0.001). g) Fluorescence microscopy images of filtrates collected from each GF/PC membrane, showing RMP passage through the membrane. h) Quantification of RMP fluorescence intensity in the filtrate as a function of parylene C deposition (*n* = 3, Student's *t*‐test: ^***^ *p* <0.001).

SEM imaging (**Figure** **3**a) and porosity analysis (Figure 3b) were performed to investigate the effect of varying the amount of parylene C on the porosity gradient formation. The SEM images show that as the level of deposition increases (0–4 g), the top surface exhibits a marked reduction in the porosity, with substantial pore closure at 4 g. In contrast, the bottom surface maintains relatively large pores, even at higher deposition levels, owing to the shielding by the internal structure of the membrane.

Quantitative porosity analysis confirmed these trends (Figure 3b). The top surface porosity decreased from 28% (0 g) to 3% (4 g), whereas the bottom surface showed a more gradual decline from 28% to 18%. This asymmetric reduction reflects the differences in parylene C thickness across the membrane, as further illustrated in Figure S1 (Supporting Information). These results confirm the successful formation of a vertical porosity gradient in response to asymmetric parylene C deposition. However, the excessive deposition (e.g., 4 g) severely reduces the top‐surface porosity, compromising the liquid permeability and overall membrane performance.^[^ ^19^, ^20^, ^21^ ^]^

Cross‐sectional SEM images of original GF and GF/PC with increasing parylene C deposition amounts (0.5–4 g) are presented in Figure 3c. The original GF exhibited a uniform fibrous structure with large and interconnected pores spanning the entire thickness of the membrane. Upon deposition of parylene C, a gradual reduction in porosity was observed, particularly at the top surface. This top‐sided densification became more pronounced with increasing parylene C amounts (0.5–4 g), whereas the bottom side retained relatively open pores. However, under the 4 g deposition condition, excessive parylene accumulation led to the near‐complete blockage of pore structures from the top to the middle region. Therefore, 2 g was selected as the optimal deposition condition, as it achieved a well‐defined porosity gradient while preserving sufficient porosity for downstream applications.

### 2.4 Effect of Parylene C Deposition on Filtration Efficiency and Hydrophilic Patterning

Porosity gradient structures are widely recognized for their ability to sequentially filter particles based on size, thereby improving the overall particle filtration efficiency.^[^ ^19^ ^]^ Because the pristine GF membrane was fully hydrophilic and lacks any porosity or wettability gradient, it offers no size‐selective filtration of 1–5 µm red fluorescent microparticles (RMPs). For example, when 10 µL of the RMP suspension was applied, it wicked through the mat in <0.1 s, and the large pore throats (>3 µm) allowed essentially 90% of the particles to pass (data not shown). As this baseline offers no additional mechanistic insight, we focused our analysis on the parylene C modified variants instead. In these membranes, the parylene deposition followed by masked O_2_ plasma treatment (Figure 3d) introduced a vertical hydrophilic path that establishes the directional capillary interface required for controlled liquid transport and particle retention.

Figure 3d–h depicts the relationship between the parylene C deposition condition (0.5–4 g) and filtered volume, as well as the particle filtration ability. To quantify these effects, membranes were inverted and 10 µL of PBS containing RMPs was applied to the large porosity side of samples prepared with varying parylene C deposition (Figure 3d). As the parylene C deposition increased from 0.5 to 2 g, the volume of filtered liquid increased from 3.4 to 8.1 µL (Figure 3e,f). However, at the highest deposition condition (4 g), excessive reduction in porosity prevented any liquid from passing through the membrane. Interestingly, despite the overall reduction in the membrane porosity with increasing parylene C deposition, the optimal porosity gradient condition at 2 g coating exhibited the highest filtered volume. This suggests that forming a porous gradient can improve liquid transport capacity following oxygen plasma treatment.

Fluorescence microscopy (Figure 3g) and fluorescence intensity analysis of the filtrate (Figure 3h) were performed to quantify particle separation performance. At 0.5 and 1 g deposition conditions, a large number of fluorescent particles passed through the membrane, resulting in elevated fluorescence intensities in the filtrate (50 and 11 a.u., respectively). In contrast, the 2 g condition effectively blocked particle passage, with minimal fluorescence detected (1.4 a.u.), confirming efficient separation at the optimized deposition level.

Additional observations from Figure S2 (Supporting Information) reveal the effect of parylene C deposition on the fidelity of hydrophilic pattern formation during O_2_ plasma treatment. At lower deposition levels (0.5–1 g), the hydrophilic patterns extended beyond the mask area on both the top and bottom surfaces, likely because of O_2_ plasma diffusion. At 2 g, the pattern size on matched the mask diameter (2.5 mm), indicating precise patterning. In contrast, deposition of 4 g impeded the O_2_ plasma access, resulting in no distinct pattern formation on the bottom surface. These results further support 2 g as the optimal deposition quantity for achieving both a porosity gradient and well‐defined hydrophilic regions.

### 2.5 Optimization of O_2_ Plasma Treatment for Self‐Pumping Induced by Wettability Gradient Formation

**Figure 4:**
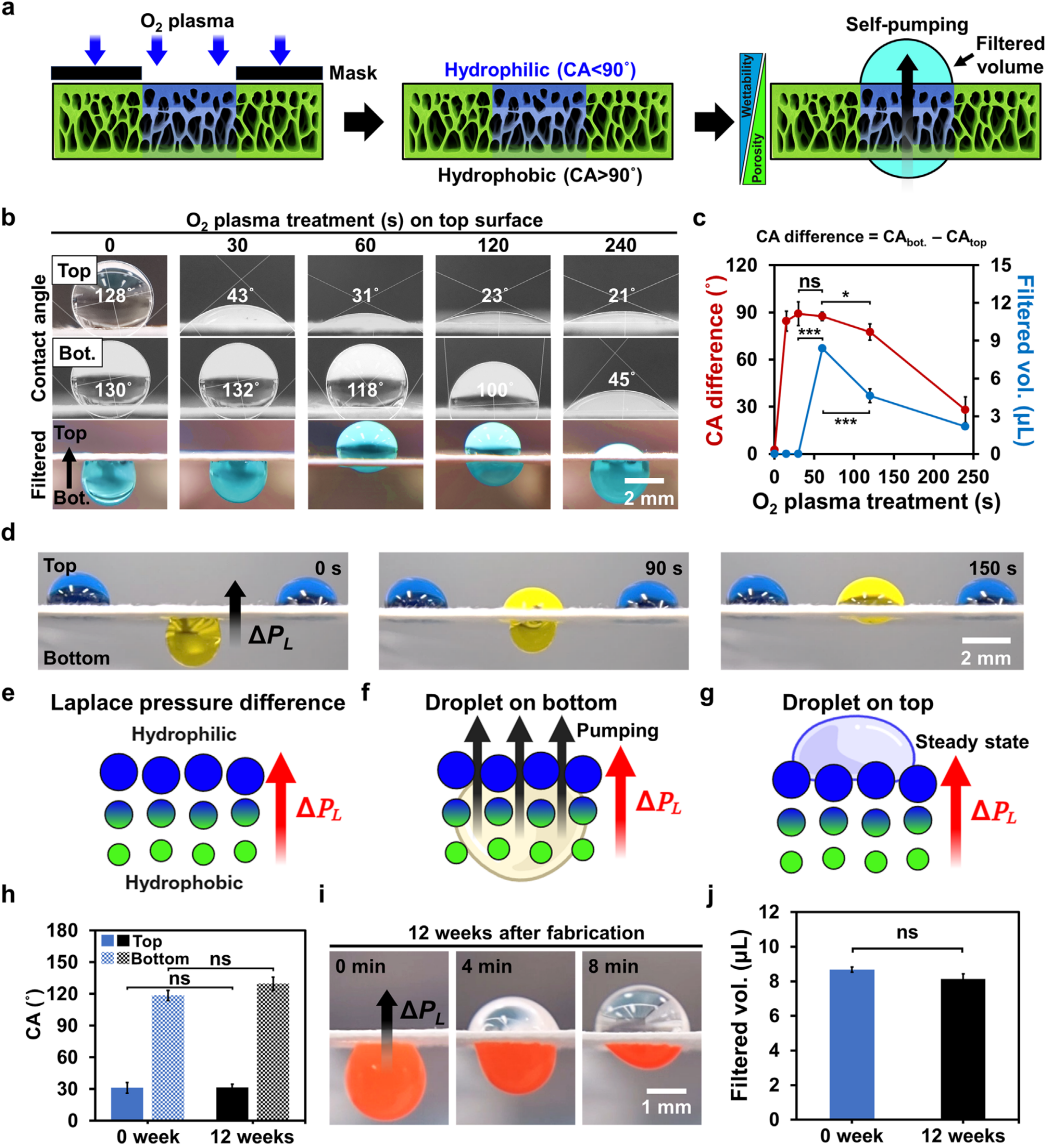
Characterization of wettability gradient induced by O_2_ plasma treatment and evaluation of long‐term self‐pumping performance. a) Schematic illustrating the formation of a wettability gradient by O_2_ plasma treatment and the evaluation of self‐pumping‐driven filtration performance. b) Water contact angle (CA) images of the top and bottom surfaces of membranes treated with O_2_ plasma for different durations (0, 30, 60, 120, and 240 s), along with corresponding images of filtered liquid volumes. c) CA differences between the bottom and top surfaces (CA difference = CA_bot._ – CA_top_) and corresponding filtered volumes as functions of the O_2_ plasma treatment time (*n* = 3, Student's *t*‐test: ns *p* >0.05, ^*^ *p* <0.05, ^***^ *p* <0.001). d) Images showing anti‐gravity self‐pumping behavior in the Janus membrane treated with O_2_ plasma for 60 s. The top droplet was stained blue and the bottom droplet yellow to visualize directional fluid transport. e) Schematic illustrating the Laplace pressure difference (Δ*P_L_*) generated by the wettability gradient across a GF/PC/O_2_ membrane. f,g) Droplet behavior on bottom and top surfaces, respectively: f) Droplet placed on the hydrophobic bottom surface was pumped upward due to the Laplace pressure difference. g) Droplet on the hydrophilic top surface remained stationary. h) CA measurements after 12 weeks of vacuum storage, demonstrating stable retention of wettability (*n* = 3, Student's *t*‐test: ns *p* >0.05). i) Time‐lapse images of self‐pumping behavior and red fluorescent particle separation after 12 weeks of vacuum storage, confirming preserved functionality. j) Filtered volumes before and after 12 weeks of vacuum storage, demonstrating consistent self‐pumping performance (*n* = 3, Student's *t*‐test: ns *p* >0.05).

To create a wettability gradient, the top surface of the GF/PC membrane was treated with O_2_ plasma using a circular mask (2.5 mm in diameter) (**Figure** **4**a). The resulting GF/PC/O_2_ membrane exhibited a Janus character, with a hydrophilic top surface (CA <90°) and a hydrophobic bottom surface (CA >90°). When liquid was introduced to the hydrophobic bottom side, it spontaneously traveled upward to the hydrophilic region, enabling self‐pumping. The amount of filtered liquid transported to the top surface was used to determine the optimal plasma treatment duration.

As shown in Figure 4b, without plasma treatment (0 s), both the top and bottom surfaces showed hydrophobic CA of ≈130°, and no liquid movement was observed. At 30 s of plasma treatment, the top surface was hydrophilic (CA = 43°) while the bottom surface was hydrophobic (CA = 132°). At 60 s, the top surface became more hydrophilic (CA = 31°), while the bottom surface showed a slight reduction in hydrophobicity (CA = 118°). For longer plasma treatments (≥120 s), the top surface was further hydrophilized (CA = 21°), and the bottom surface continued to lose hydrophobicity (CA = 100°). At 240 s, both surfaces became hydrophilic (top CA = 21°, bottom CA = 45°).

This progressive shift in surface wettability indicated that hydrophilicity was first restored on the top surface and subsequently on the bottom surface. This behavior was attributed to the formation of hydrophilic functional groups via plasma treatment (Figure 2d). It suggested that the plasma penetrated along the porous structure, gradually modifying the bottom surface. Therefore, optimizing plasma treatment duration was necessary to achieve efficient fluid transportation.

To investigate this, the filtration volumes at each treatment duration were measured (Figure 4b). The 60 s condition resulted in the highest filtered volume from bottom to top. To better understand this trend, the relationship between the CA difference (CA difference = CA_bot._ – CA_top_) and the filtered volume was analyzed (Figure 4c). Although the CA differences at 30 s (89°) and 60 s (87°) were similar, only the 60 s condition enabled substantial fluid flow (8.4 µL), whereas the 30 s condition did not. This discrepancy is explained in Figure S3 (Supporting Information), which shows that the hydrophilic region at 30 s extended only partially through the membrane, while at 60 s, it penetrated the full thickness. At 120 s, the CA difference narrowed to 77°, and the filtered volume was reduced to 4.6 µL. At 240 s, the CA difference dropped sharply to 28°, and the filtered volume was minimal (2.1 µL), indicating substantially weakened self‐pumping. This correlates with the loss of vertical wettability contrast and the onset of lateral diffusion of the plasma effect across the membrane surface, as shown in Figure S3 (Supporting Information). These findings indicate that both a sufficient CA difference and complete vertical penetration of the hydrophilic region are essential to achieve effective self‐pumping through a wettability gradient.

This difference in top and bottom surface hydrophilicity was also reflected in the O 1s peak analysis, where a clear distinction was observed under the optimal 60 s condition, but no significant difference was found under the untreated (0 s) or overtreated (240 s) conditions (Figure S4, Supporting Information). A similar trend in wettability‐driven fluid transport has been reported in previous studies on membranes with wettability gradient,^[^ ^2^, ^3^, ^22^ ^]^ reinforcing the importance of controlled surface contrast. These findings demonstrate that 60 s of O_2_ plasma treatment established the most effective wettability gradient for gravity‐independent fluid transport.

The self‐pumping behavior of the membrane was evaluated under anti‐gravity conditions, where liquid applied to the lower (bottom) surface was transported upward against gravity due to the optimized porosity and wettability gradients (2 g parylene C, 60 s O_2_ plasma) (Figure 4d; Movie S1, Supporting Information). Yellow liquid placed on the hydrophobic bottom surface migrated upward to the hydrophilic top surface within 150 s, whereas blue liquid on the top surface remained stationary. This unidirectional liquid transport is presumably driven by Laplace pressure differences (Δ*P_L_*) which arise from variations in surface wettability, as previously reported.^[^ ^1^, ^3^, ^4^, ^22^ ^]^ Laplace pressure in each surface of the Janus membrane can be obtained from the Young‐Laplace equation (Equation 1):

(1) $$P_{L}=\frac{4\gamma \cos\theta }{d}\;$$

where, *P_L_* represents the Laplace pressure (Pa), γ is the surface tension of water (N/m), θ is the CA of water with the surface (°), and d is the pore diameter (m).^[^ ^3^ ^]^

In the present membrane, the top surface (hydrophilic) exhibited a higher Laplace pressure than the bottom surface (hydrophobic), establishing a pressure gradient that drives the liquid transport upward (Figure 4e).^[^ ^1^, ^3^, ^22^ ^]^ Accordingly, a larger CA difference between the two surfaces results in a steeper Laplace pressure gradient, thereby enhancing the driving force for fluid transport. Figure 4f illustrates that liquid placed on the hydrophobic bottom surface is pumped upward due to this Laplace pressure gradient. In contrast, as shown in Figure 4g, liquid placed on the hydrophilic top surface remains stationary, as it opposes the direction of the pressure gradient. Reversing the membrane orientation also reverses the flow direction, enabling gravity‐assisted pumping (Movie S2, Supporting Information). To emphasize the importance of gradient alignment, we intentionally misaligned the porosity and wettability gradients. This misalignment reduced the filtered volume by approximately half compared with the aligned configuration (Figure S5, Movie S3, Supporting Information). Previous studies have demonstrated that the self‐pumping behavior of Janus membranes arises from a wettability gradient or in combination with a porosity gradient.^[^ ^23^, ^24^, ^25^ ^]^ Our results further show that the alignment of the wettability and porosity gradients synergistically contributes to enhancing the self‐pumping effect.

Moreover, the Janus membrane maintained its dual‐gradient structure and functionality after 12 weeks of vacuum storage, with preserved CA values (Figure 4h), self‐pumping behavior (Figure 4i), and filtered volume (Figure 4j), thereby demonstrating long‐term durability. In addition to long‐term vacuum storage, we also evaluated the short‐term aqueous stability of the membrane. As shown in Figure S6 (Supporting Information), the Janus membrane retained its separation ability and self‐pumping function after 24 h storage in deionized water (DW), indicating its operational robustness under wet conditions. The PPP treatment was also successfully applied to cellulose‐based substrates, such as Whatman 4 cellulose filter paper, which exhibited similar anti‐gravity fluid transport (Figure S7, Supporting Information). Unlike high‐temperature techniques required for fabricating metal‐based dual‐gradient Janus systems,^[^ ^26^ ^]^ this low‐temperature approach enables scalable fabrication across diverse porous materials, offering promising opportunities for next‐generation fluidic applications.

### 2.6 Plasma Separation using Dual‐Gradient Janus Membrane

**Figure 5:**
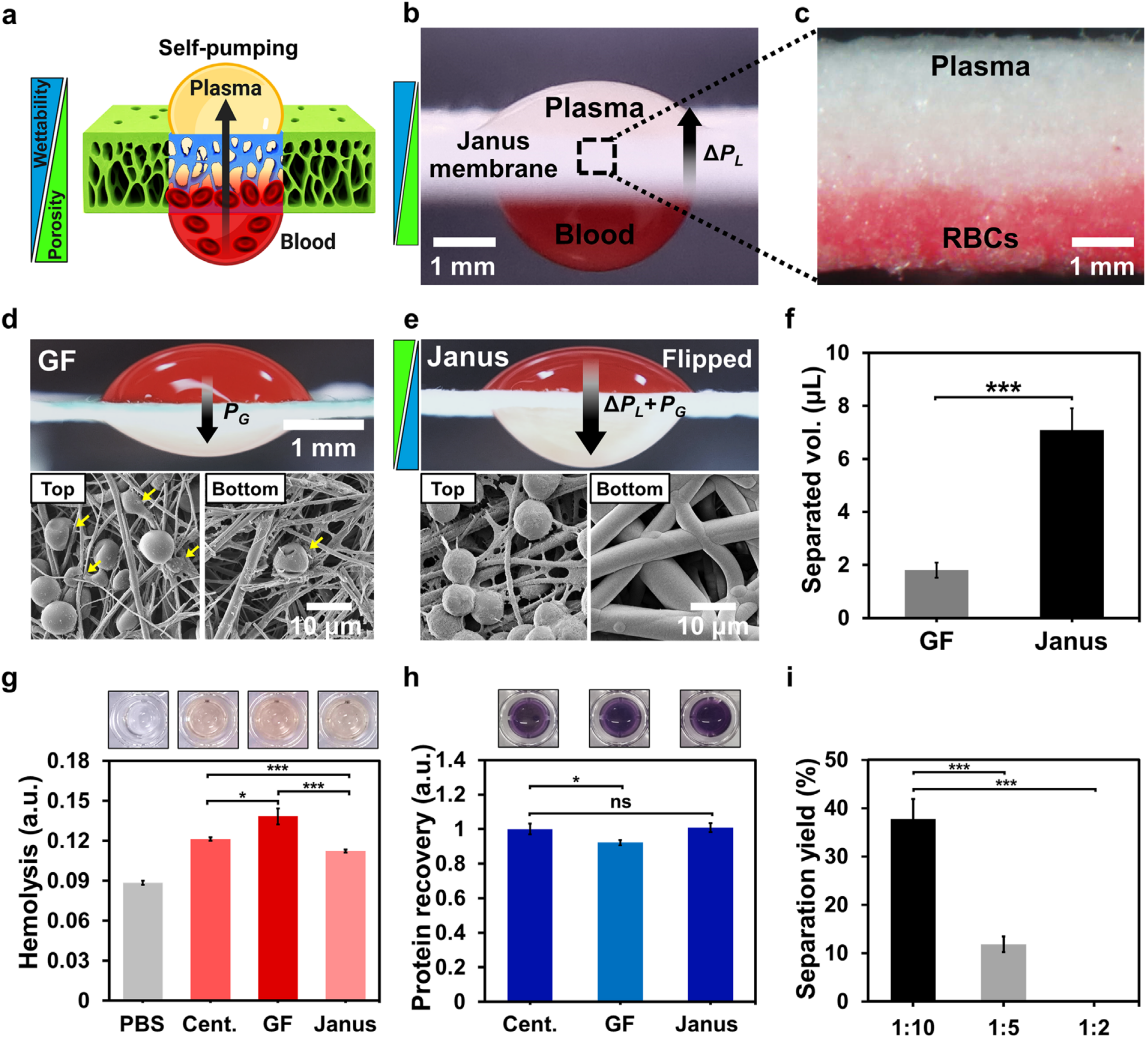
Plasma separation using dual‐gradient Janus membranes. a) Schematic of plasma separation in an anti‐gravity configuration. When diluted defibrinated blood is applied to the hydrophobic bottom side, RBCs are retained in the lower porous region, while plasma is drawn upward to the hydrophilic top via a Laplace pressure gradient. The blue and green boxes indicate the directions of the wettability (hydrophobic to hydrophilic) and porosity (smaller to larger) gradients, respectively. b) Images of Janus membranes during plasma separation, showing plasma accumulation on top and blood retained at the bottom (10 min, 1:10 diluted blood). (Arrow indicate direction of Laplace pressure gradient). c) Cross‐sectional view of the membrane post‐separation, showing RBCs selectively trapped in the red‐stained bottom zone and clear plasma in the upper region. d,e) Comparison of plasma separation performance between d) a standard GF membrane driven by gravity‐induced pressure (*P*_G_) and e) a flipped Janus membrane driven by both the Laplace pressure gradient (Δ*P_L_*) and gravity‐induced pressure (*P*_G_). SEM images (×5000) of both surfaces show ruptured RBCs in the GF (yellow arrows), whereas the Janus membrane retains the RBCs intact and inhibits their leakage. To visualize the RBCs on the membrane, a pretreatment protocol was performed prior to imaging, as detailed in Section 2. f) Quantitative comparison of plasma separation yield between the Janus and GF membranes (*n* = 6, Student's *t*‐test: ^***^ *p* <0.001). g) Absorbance of plasma at 562 nm, reflecting the extent of hemolysis. The Janus membrane shows the least hemolysis when compared with those of centrifugation (1000× g, 5 min) and the bare GF membrane (*n* = 3, Student's *t*‐test: ^*^ *p* <0.05, ^**^ *p* <0.01, ^***^ *p* <0.001). h) Protein recovery via BCA assay shows comparable recovery for Janus and centrifuge methods, with reduced recovery in the GF (*n* = 3, Student's *t*‐test: ns *p* >0.05, ^*^ *p* <0.05). i) Plasma separation yields at different blood dilution ratios (1:10, 1:5, and 1:2) (*n* = 6, Student's *t*‐test: ^***^ *p* <0.001).

To evaluate the performance of Janus membranes, plasma separation experiments were conducted using defibrinated sheep blood diluted 1:10 with PBS (**Figure** **5**). In the anti‐gravity configuration, blood was applied to the bottom surface of the membrane, where the red blood cells (RBCs) were trapped in the lower porous region, whereas the plasma was self‐pumped to the top surface (Figure 5a–c). A 2 µL glucose‐sensitive reagent applied to the collected plasma produced a visible color change from yellow to green, confirming the presence of glucose.^[^ ^8^, ^27^ ^]^ In contrast, the reagent applied to PBS showed no color change, validating the ability of the membrane to remove cellular interference for direct enzymatic detection (Movie S4, Supporting Information).

To enable a direct comparison under identical gravity‐driven conditions, the Janus membrane was flipped so that plasma separation occurred in the downward direction. This adjustment was necessary because the standard GF membrane lacks a wettability gradient and therefore cannot achieve plasma separation in the anti‐gravity direction. Compared to the standard GF membrane, the Janus membrane achieved a fourfold increase in plasma yield under gravity‐driven conditions (Figure 5d–f), which can be attributed to the combined effect of Laplace pressure gradient (Δ*P_L_*) and gravity‐induced pressure (*P*_G_) generated by the dual‐gradient structure. Under the same conditions, no plasma transport was observed in the commercial plasma separation membrane (PSM) (Figure S8, Supporting Information). SEM imaging revealed significant RBC rupture in the GF membrane due to its sharp fibers, whereas the Janus membrane preserved RBC morphology and prevented leakage into the plasma side. This improvement can be attributed to the parylene C coating, which smooths the fiber surfaces and reduces porosity, thereby enhancing filtration performance.

Hemolysis and protein recovery were assessed to evaluate whether the plasma separated using the Janus membrane maintained comparable biochemical quality (Figure 5g,h). Hemolysis was lowest in plasma collected from the Janus membrane (0.11), followed by plasma obtained via centrifugation (0.12) and the GF membrane (0.14), likely due to the gentler self‐pumping mechanism of the Janus membrane. Protein recovery, as measured using the BCA assay, was comparable between the Janus membrane and centrifugation methods, whereas the GF membrane showed a significant reduction, likely due to incomplete plasma extraction.

The separation performance of the Janus membrane was further evaluated across different blood dilution ratios (Figure 5i). Plasma yield was 37% at a 1:10 dilution but decreased to 12% at 1:5. No plasma was recovered at a 1:2 dilution, indicating that the capillary‐driven force of the Janus membrane is insufficient to process highly viscous, undiluted blood.

Overall, these findings highlight the critical role of the porosity gradient in enabling efficient RBC filtration and plasma transport. The asymmetric structure of the Janus membrane is well suited for advanced filtration systems such as plasma separation membranes, which are widely used to isolate plasma and cellular components.^[^ ^8^, ^28^, ^29^ ^]^ The plasma quality achieved using the Janus membrane was comparable to that of centrifugation, particularly with respect to hemolysis and protein recovery,^[^ ^30^, ^31^ ^]^ with the added advantage of operating without external force. Unlike conventional plasma separation devices that rely on gravity‐driven flow and require large blood volumes,^[^ ^32^, ^33^ ^]^ our membrane enables efficient separation from fingertip‐scale blood samples. Additional evaluations such as complement activation or coagulation analysis may be considered in future studies to further establish blood compatibility, particularly for clinical or therapeutic applications. Moreover, further optimization is needed to improve separation performance with undiluted whole blood.

## 3 Conclusion

In this study, we developed a scalable, low‐temperature PPP treatment strategy that transforms conventional porous membranes into dual‐gradient Janus structures with asymmetric wettability and porosity. By integrating asymmetric parylene C deposition with selective O_2_ plasma treatment, the PPP treatment enables simultaneous and spatially aligned control of both parameters within a single membrane substrate. This anisotropic design facilitates capillary‐driven, gravity‐independent fluid transport, thereby eliminating the need for external pumps. The multifunctionality of the resulting membrane was demonstrated by efficient plasma separation from microliter‐scale whole blood samples with minimal hemolysis. These findings underscore the potential of the PPP‐treated Janus membrane for point‐of‐care (POC) diagnostic applications, particularly in settings that require passive blood separation from small‐volume samples without active pumping systems.

Notably, the low‐temperature, solvent‐free nature of the PPP treatment allows compatibility with diverse substrates including cellulose and glass fiber making it well suited for large‐area, scalable production. Looking ahead, the ability to engineer dual‐gradient membranes via the PPP treatment holds strong promise for advancing POC diagnostic technologies, particularly in vertical‐flow assays and passive plasma separation platforms. Collectively, these features position PPP treatment as a versatile and practical platform for next‐generation membrane development tailored to the needs of POC applications.

## 4 Experimental Section

### Materials

Dichloro‐di‐p‐xylylene (parylene C) was purchased from Daisan Kasei Co., Ltd. (Tokyo, Japan). Whatman grade 4 cellulose filter paper and Whatman glass fiber (GF/A) were purchased from Whatman International Ltd. (Maidstone, UK). Plasma separation membranes (PSMs) (Vivid GR), made of polysulfone, were purchased from Pall Life Sciences (Ann Arbor, MI, USA). D‐(+)‐glucose, glucose oxidase (GOx), and 4‐aminoantipyrine (4‐AAP) were purchased from Sigma–Aldrich. Horseradish peroxidase (HRP) was obtained from Toyobo Co., Ltd. (Osaka, Japan). N‐Ethyl‐N‐(2‐hydroxy‐3‐sulphopropyl)‐3,5‐dimethylaniline sodium salt monohydrate (MAOS) was purchased from Dojindo (Rockville, MD, USA). Phosphate‐buffered saline (PBS, pH 7.4) and bicinchoninic acid (BCA) protein assay kit were purchased from Thermo Fisher Scientific. Masking tape (LB‐PS‐02/A4 20) was purchased from Samhyup Tape (Gunpo, Korea).

### Asymmetric CVD of Parylene C on Porous Membrane to Form a Porosity Gradient

To form a porosity gradient in the porous membrane, the bottom surface of the porous membrane was tightly affixed to the deposition plate (diameter: 15 mm) of a parylene deposition system (LAVIDA 110, Femto Science Ltd., Hwaseong, Korea), and all edges were sealed using masking tape to ensure that parylene C permeated and deposited exclusively on the top surface of the porous membrane. The deposition plate was placed on a rotating stage inside the deposition chamber, which maintained RT and operated at a speed of 3 rpm during the deposition process. Subsequently, 2 g of parylene C dimer (dichloro‐di‐p‐xylylene) was loaded into the vaporizer, and the chamber was sealed to initiate the deposition process. The deposition of parylene C was performed in four sequential steps under a vacuum of <4 Pa (30 mTorr): 1) pre‐heating the evaporation chamber until it reached 130 °C to prevent the rapid evaporation of parylene C; 2) evaporation of parylene C powder at 160 °C, generating vapor‐phase monomers; 3) pyrolysis at 690 °C, where the vaporized parylene C dimer is thermally decomposed in the pyrolysis furnace into highly reactive free radicals; and 4) deposition and polymerization at RT, where the reactive species condensed and polymerized onto the porous membrane in the deposition chamber. The evaporation process required 2 h to fully evaporate 2 g of parylene C (evaporation rate: 1 g h^−1^). After the deposition process, the masking tape was carefully removed to expose the full membrane for subsequent treatments.

### O_2_ Plasma Treatment to Form a Wettability Gradient

To form a wettability gradient, both the top and bottom surfaces of the porous membrane were masked using a patterned masking tape. The masking tape on the top surface featured a specific pattern to allow selective exposure to O_2_ plasma, whereas the bottom surface was fully covered to prevent O_2_ plasma exposure. Following the masking, the membrane underwent O_2_ plasma treatment using a plasma system (Cute, Femto Science Ltd.) under the following conditions: 60 W power, 50 mTorr pressure, for 1 min. After the O_2_ plasma treatment, the masking tapes were carefully removed to reveal the functionalized membrane.

### FE‐SEM and EDS Mapping for Structural and Elemental Analysis

FE‐SEM imaging and EDS mapping analyze were conducted to observe the structural and elemental changes in each fabrication stage—untreated GF, parylene C‐coated GF (GF/PC), and O_2_‐plasma‐treated GF/PC (GF/PC/O_2_)—enabling a comprehensive evaluation of the gradient formation process. For this purpose, FE‐SEM equipped with EDS (JSM7500F; JEOL Ltd., Tokyo, Japan) was utilized for both surface and cross‐sectional imaging. Before the imaging, the membranes were cut using a surgical blade and mounted securely on the sample holder. All samples were coated with a thin layer of iridium (Ir) to enhance the conductivity. The FE‐SEM equipment was operated at an accelerating voltage of 15 kV with a working distance of 8 mm. To prepare red blood cells (RBCs) on membranes for FE‐SEM imaging after plasma separation, the samples were rinsed with PBS and fixed in 2.5% glutaraldehyde (PBS, 37 °C, 30 min). The fixed membranes were then dehydrated using a graded ethanol series (25–100%, 15 min each).^[^ ^34^ ^]^

### XPS Analysis for Surface Chemical Characterization

XPS analysis was conducted to confirm the surface modification and chemical composition of the modified GF. The GF samples were placed on a glass slide, and the XPS spectra of the surfaces were obtained using an XPS analyzer (ESCALAB™ 250, Thermo Fisher Scientific).

### Fiber Thickness and Porosity Measurements

The SEM images of the top and bottom surfaces of the GF samples under different parylene C coating conditions were imported into ImageJ (NIH, USA). For measuring the fiber thickness, at least 30 fibers were randomly selected from each surface and measured to determine the thickness distribution. For the porosity analysis, the SEM images were converted into a binary format to distinguish the pore regions from the membrane fibers.^[^ ^35^, ^36^ ^]^ The threshold range was uniformly adjusted to 75–85% to select only the pore areas, thus minimising background noise.^[^ ^37^ ^]^ The particle analysis function of ImageJ was used to calculate the pore area percentage. All images were acquired under identical magnification and field of view to ensure comparability. This image‐based approach was previously shown to yield porosity values comparable to those obtained by a helium‐gas pycnometer, supporting its validity for characterizing porous fibrous membranes.^[^ ^34^ ^]^

### Stereomicroscopy for Membrane Cross‐Section Analysis

The membrane cross‐sections were examined using images obtained with a stereomicroscope (SMZ1500, Nikon, Tokyo, Japan) equipped with a charge‐coupled device camera.

### Water Contact Angle Measurements

Water contact angle measurements were performed using the contact angle plug‐in of ImageJ.^[^ ^38^ ^]^ A 5 µL water droplet was placed on the membrane surface, and an image was captured within 5 s using a Galaxy S20 smartphone (Samsung, Korea) with a 10× macro lens.

### Filtered Volume Measurements

The filtered volume was quantified using membranes featuring a 2.5 mm‐diameter wettability gradient path. A 10 µL PBS droplet was applied to the hydrophobic (bottom) side, and fluid transport was allowed for 10 min until the flow ceased. The permeated liquid collected on the top surface was collected, and its weight was measured using an analytical balance to calculate the filtered volume.

### Particle Separation Test

The particle separation performance of the membranes was evaluated using PBS containing red fluorescent microparticles (RMPs, 1–5 µm diameter, excitation/emission: 575/607 nm; Cospheric, Somis, CA, USA). A 10 µL droplet of the RMP solution was applied onto the hydrophobic (bottom) side of the membrane (2.5 mm diameter). After 10 min, the filtrate was collected to measure the filtered volume, perform fluorescence imaging, and analyze the fluorescence intensity. The fluorescence images were acquired using a DeltaVision microscope (GE Healthcare, Chicago, IL, USA). Intensity analysis was performed by transferring 100 µL of the filtrate to a microtube, which was illuminated with a 570 nm LED in a photo box and imaged using a Galaxy S21 smartphone equipped with a 605 nm fluorescence filter (Edmund Optics, Barrington, NJ, USA). The grey intensity of the filtrate images was quantified using ImageJ.

### Plasma Separation Test and Evaluation of Separation Quality

Plasma separation tests were conducted using defibrinated sheep blood (KisanBio; Gunpo, Korea) to evaluate the plasma separation performance of the GF and Janus membranes. As the GF membrane lacks self‐pumping capability, the separation for both membrane types was performed under gravity‐driven conditions. In brief, blood was diluted to 1:10 with PBS, and 20 µL of the diluted blood was applied to the top surface of the membrane (2.5 mm diameter). After 10 min, the plasma transported through the membrane was collected from the bottom, weighed, and converted to volume based on the plasma density. To assess the quality of the separated plasma, hemolysis and protein recovery performances were evaluated and compared with those of a bench‐top centrifugation method (1000× g, 5 min). Hemolysis was quantified by measuring the absorbance at 576 nm (free hemoglobin)^[^ ^30^, ^39^ ^]^ using a microplate reader (Molecular Devices) with 100 µL of plasma. Protein recovery was determined using the BCA assay after incubating the plasma samples at 37 °C for 30 min and reading the absorbance at 562 nm. For downstream glucose detection, the enzymatic reagent was prepared by dissolving MAOS (1 mm), 4‐AAP (10 mm), HRP (1 mg mL^−1^), and GOx (10 mg mL^−1^) in PBS (pH 7.4).^[^ ^8^ ^]^

### Statistics

All data were expressed as the mean ± standard deviation (SD) from three or more independent experiments. The statistical significance was determined using Student's *t*‐test. The levels of statistical significance were set at ns *p*>0.05, ^*^ *p* <0.05, ^**^ *p* <0.01, and ^***^ *p* <0.001.

## Conflict of Interest

The authors declare no conflict of interest.

## Supporting information



Supporting Information

Supplemental Movie 1

Supplemental Movie 2

Supplemental Movie 3

Supplemental Movie 4

## Data Availability

The data that support the findings of this study are available in the supplementary material of this article.
