# Supplementary material for: Transforming Porous Membranes into Dual‐Gradient Janus Structures by Directional Asymmetric Modification
Source: Small Methods. 2025 Aug 7;9(9):e00839. doi: 10.1002/smtd.202500839 (PMC12464810; doi:10.1002/smtd.202500839)
Supplement: Supplementary file 1 — Supporting Information [file SMTD-9-e00839-s003.docx]

Supporting Information

**Transforming Porous Membranes into Dual-Gradient Janus Structures by Directional Asymmetric Modification**

Jaehyung Jeon, Heeseon Choi, Jinseung Bae, Gwang Myeong Seo, Jeonghun Han, Hogyun Park, and Sungsu Park*

J. Jeon, H. Choi, J. Bae, G.M. Seo, J. Han, H. Park, S. Park

School of Mechanical Engineering, Sungkyunkwan University (SKKU), Seoburo 2066, Jangan-gu, Suwon 16419, Korea

E-mail: [nanopark@skku.edu](file:///C:\Users\nanos\Downloads\nanopark@skku.edu)

**Supplementary figures**

Figure. S1 Fiber thickness at the top and bottom surfaces of parylene C-deposited glass fiber membranes (GF/PC) as a function of the deposition amount. The thickness was measured after depositing 0, 0.5, 1, 2, and 4 g of parylene C. SEM images of the top and bottom surfaces of the GF samples under different parylene C coating conditions were imported into ImageJ (NIH, USA). For the fiber thickness measurements, at least 30 fibers were randomly selected from each surface and measured to determine the thickness distribution. A minimum of 30 fibers from each surface were randomly selected and measured to determine the fiber thickness distribution.

**
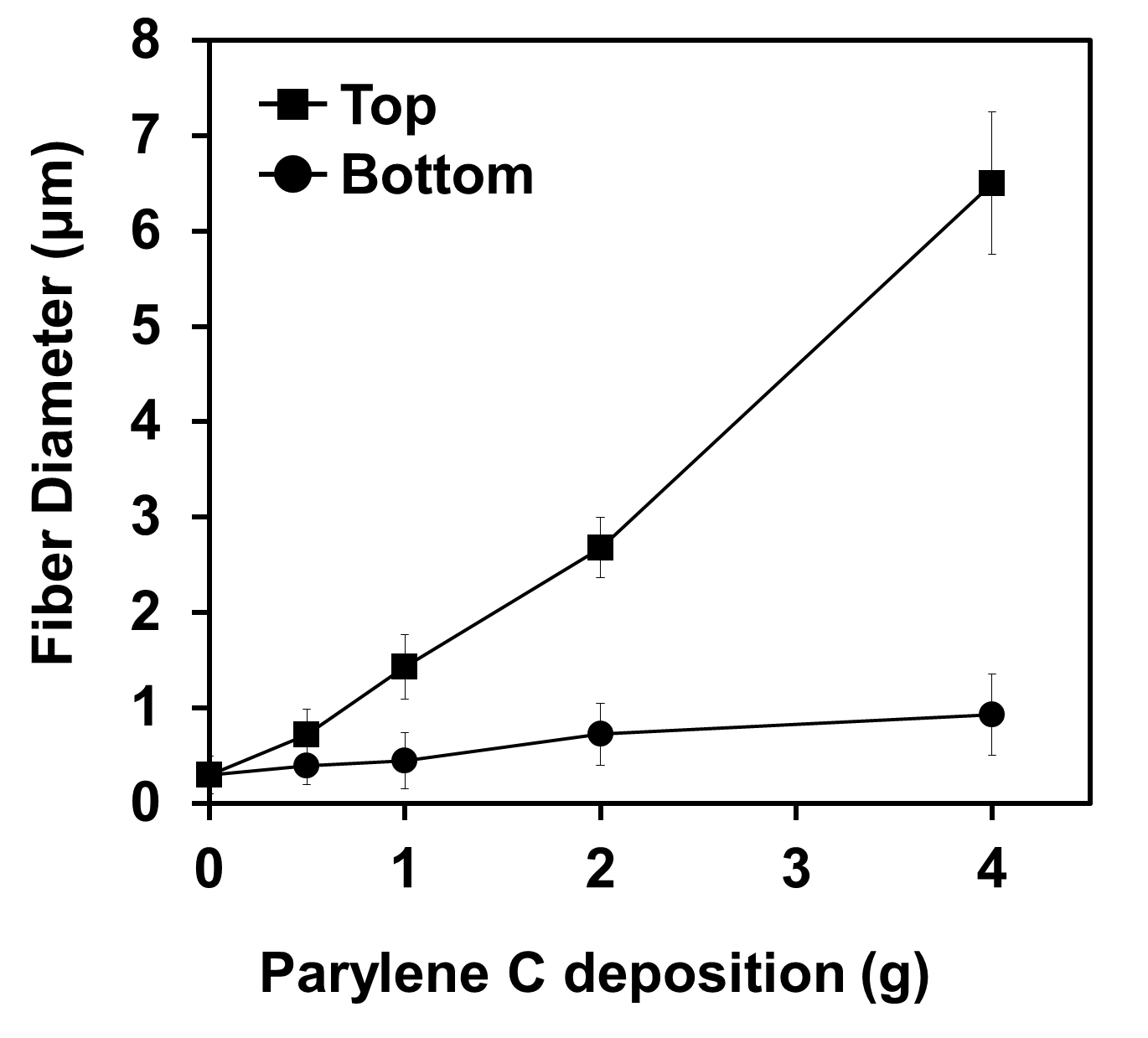
**

Figure. S2 Effect of level of parylene C deposition on the formation of hydrophilic patterns under 60 s of oxygen plasma treatment. a) Schematic of the mask pattern design. b**)** Illustration of directional oxygen plasma treatment using a patterned mask on the membrane surface. c) Top and bottom surface images of hydrophilic patterns formed on GF/PC membranes with different amounts of parylene C deposition (0.5, 1, 2, and 4 g) and treated with oxygen plasma for 60 s. d) Quantitative analysis of hydrophilic pattern diameters of top and bottom surfaces as functions of the amount of parylene C deposition.


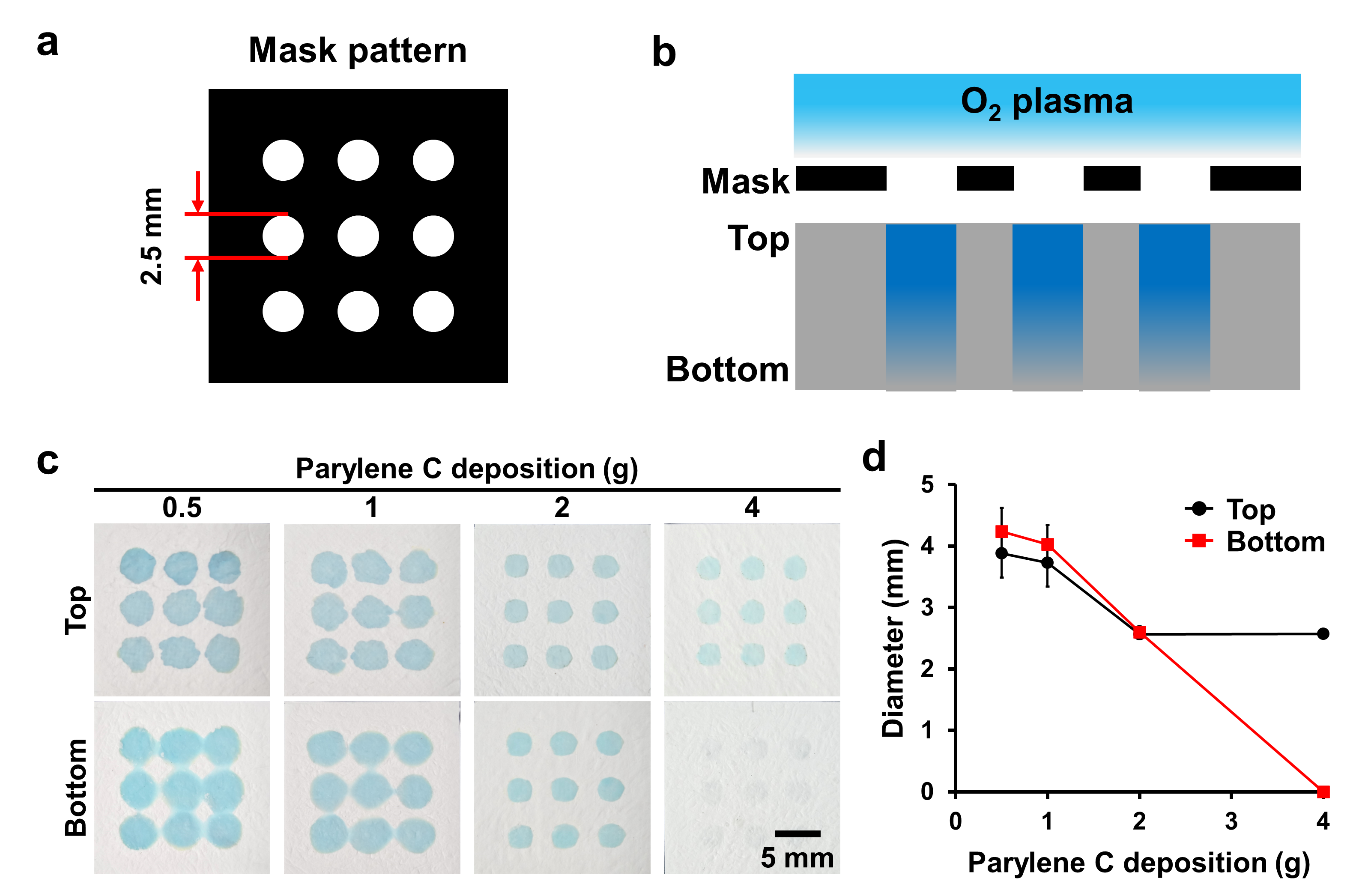


Figure. S3 Depth analysis of hydrophilic pattern formation within GF/PC for different oxygen plasma treatment durations. Hydrophilic regions were visualized by applying blue ink droplets to cross-sections of the membrane. At 30 s, the hydrophilic region extended only to the middle of the membrane. The optimal condition (60 s) resulted in a fully developed gradient that reached the bottom surface. In contrast, over-treatment (240 s) caused lateral diffusion of the hydrophilic region. The membrane cross-sections were examined using images obtained with a stereomicroscope (SMZ1500, Nikon, Tokyo, Japan) equipped with a charge-coupled device (CCD) camera.


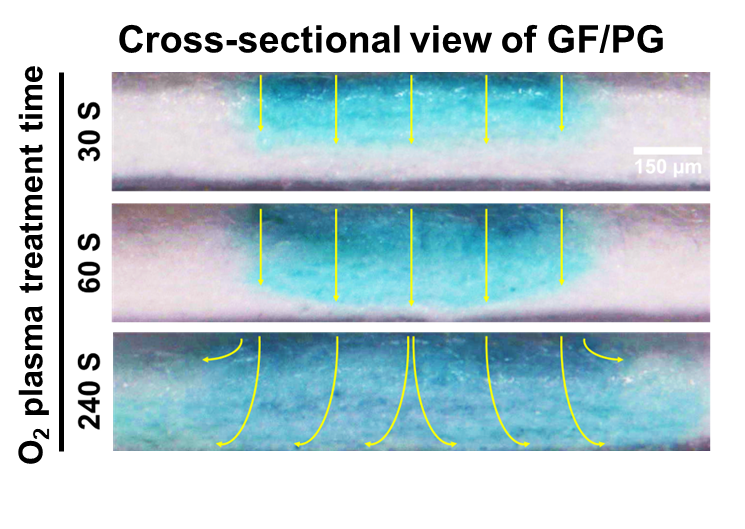


Figure. S4 O 1s XPS spectra of the top and bottom surfaces of the membrane under different oxygen plasma treatment conditions. The O 1s peak analysis reveals distinct differences in wettability after 60 s of treatment, where the top surface shows a significantly higher O 1s peak intensity than that at the bottom surface. XPS spectra of the surfaces were obtained using an XPS analyzer (ESCALAB^TM^ 250, Thermo Fisher Scientific).

**
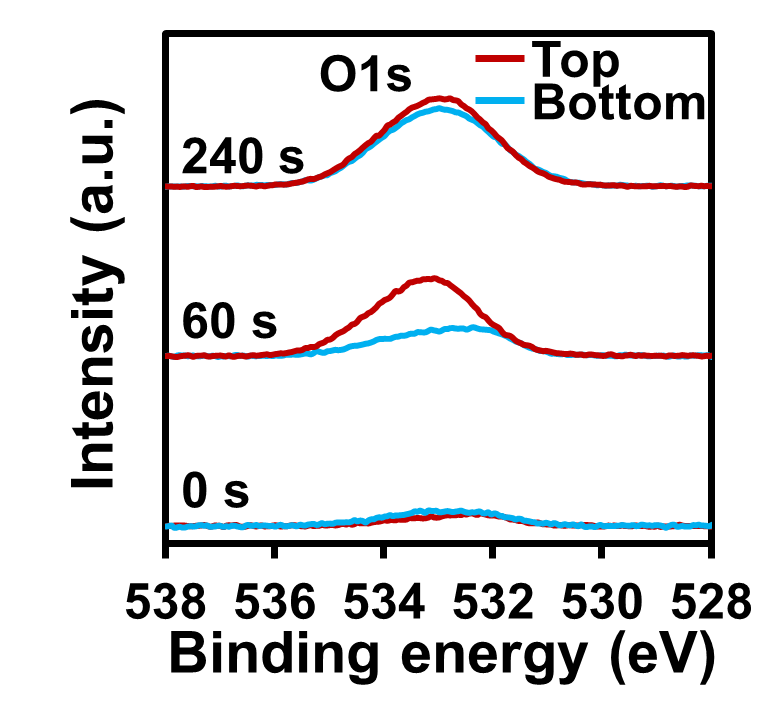
**

Figure. S5 Effect of alignment of porosity and wettability gradients. a, b) Time-lapse images comparing the self-pumping capability of membranes with a) aligned (top: small pores, hydrophilic; bottom: large pores, hydrophobic) versus b) misaligned (top: large pores, hydrophilic; bottom: small pores, hydrophobic) dual gradients. c) Quantitative comparison of filtered volume between aligned and misaligned dual gradients. The filtered volume was quantified using membranes featuring a 2.5 mm-diameter wettability gradient path with a 10 μL PBS droplet. The permeated liquid accumulated on the top surface was collected, and its weight was measured using an analytical balance to calculate the filtered volume.


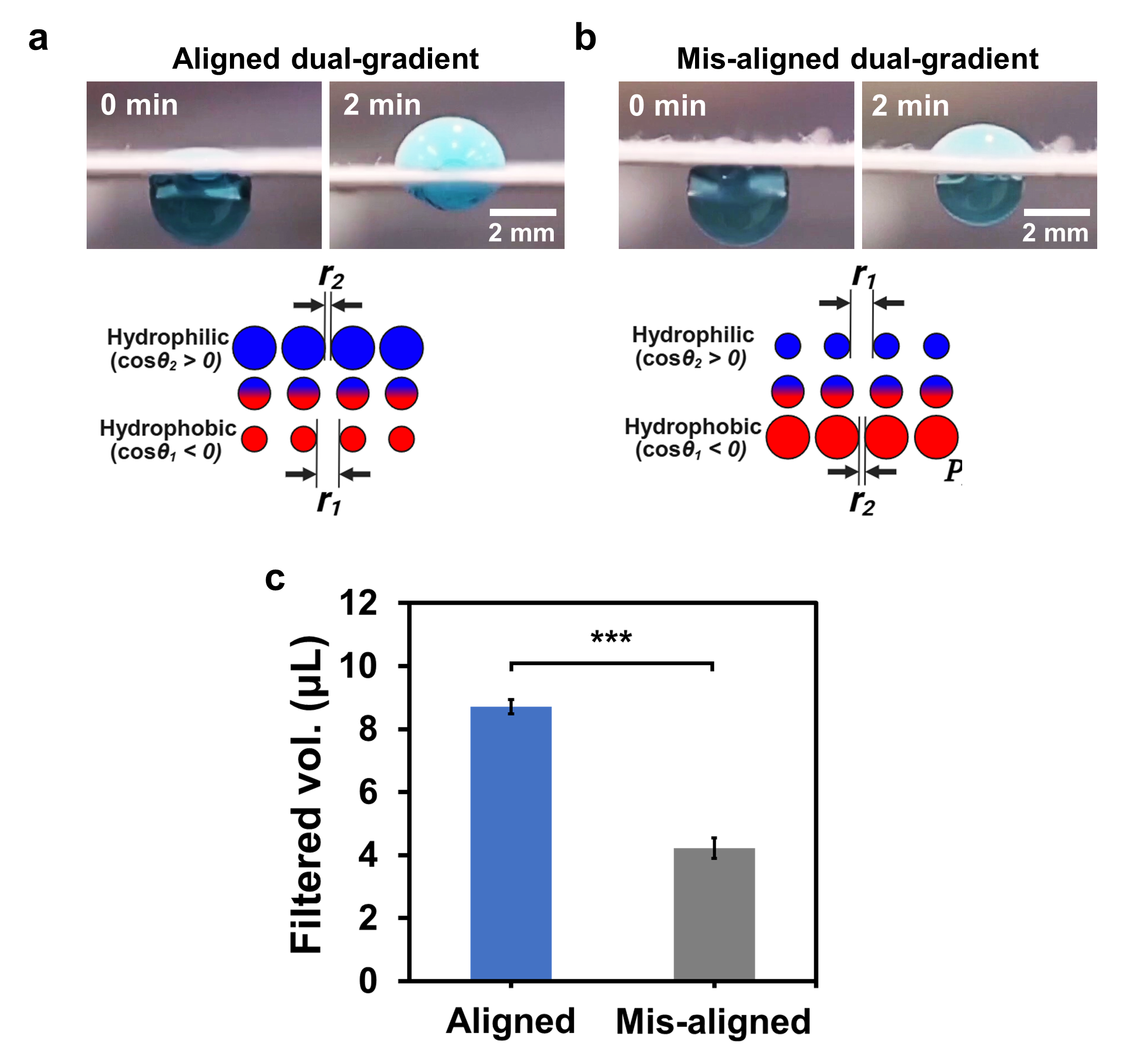


Figure. S6 Time-lapse images of self-pumping behavior and red fluorescent particle separation after 24 hours of deionized water (DW) storage, confirming preserved functionality in liquid.

**
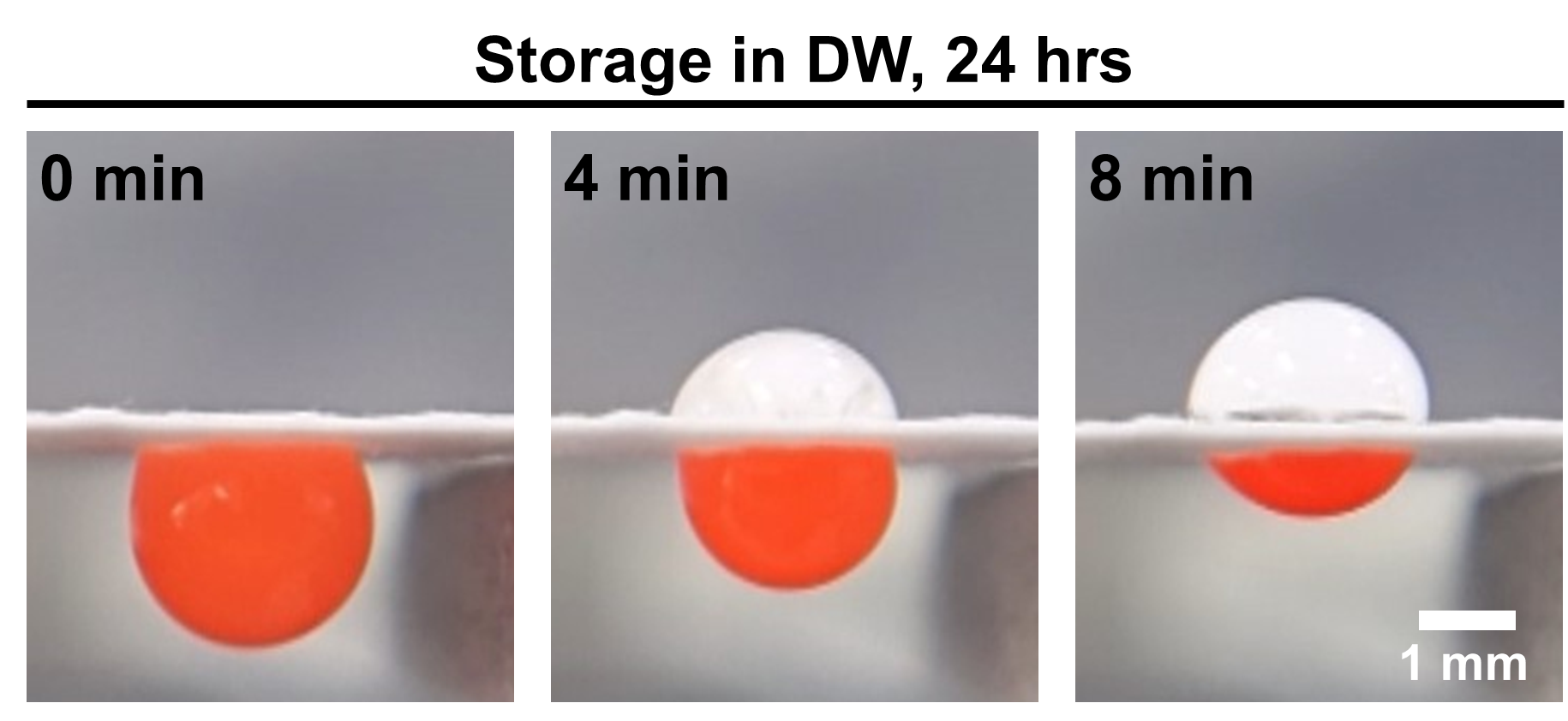
**

Figure. S7 Janus membrane formation and self-pumping capability of a Whatman 4 cellulose membrane using parylene-plasma-porous PPP treatment. a) Contact angle (CA) images of the top and bottom surfaces of a cellulose membrane coated with 2 g of parylene C and treated with oxygen plasma for varying durations (5, 10, 15, and 30 s). b) Quantitative analysis of CA as a function of plasma treatment time. c) Time-lapse images showing self-pumping behavior of Janus membrane in the anti-gravity direction under the optimal 15 s plasma treatment condition.

**
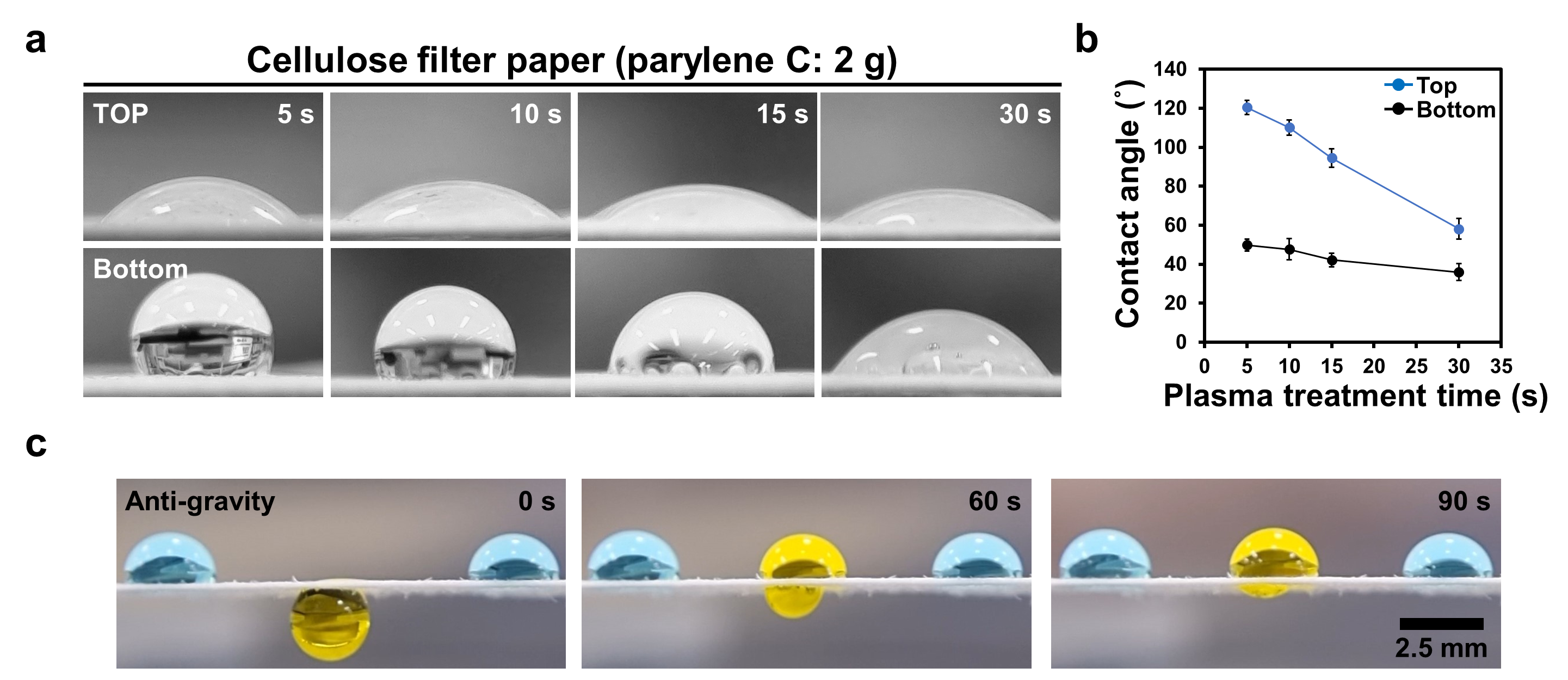
**

Figure. S8 Gravity-driven plasma separation using a commercial Vivid™ plasma separation membrane (PSM). The image shows a blood droplet on the surface of the Vivid™ PSM, with no visible plasma transport to the bottom layer. Defibrinated sheep blood (KisanBio; Gunpo, Korea) was diluted 1:10 with PBS, and 20 μL of the diluted blood was applied to the top surface of the PSM for 10 min.

**
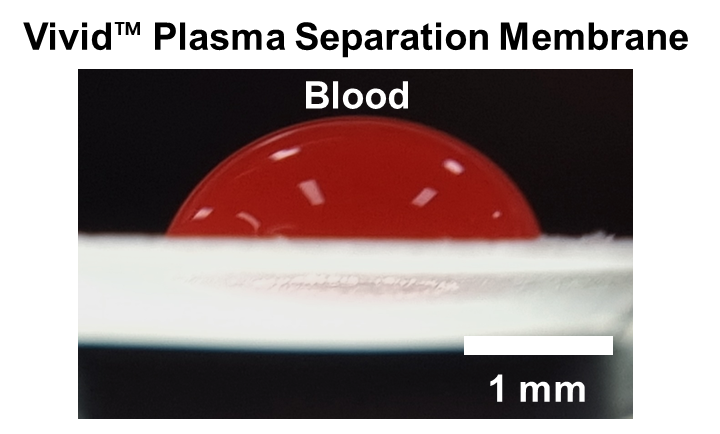
**

**Supplementary Movie:**

Movie S1. Gravity-assisted pumping of Janus membrane

Movie S2. Anti-gravity pumping of Janus membrane

Movie S3. Effect of porosity gradient and wettability gradient alignment on self-pumping performance

Movie S4. Blood plasma separation in the anti-gravity direction using Janus membrane
